# Effect of Maternal Dietary Redox Levels on Antioxidative Status and Immunity of the Suckling Off-Spring

**DOI:** 10.3390/antiox10030478

**Published:** 2021-03-17

**Authors:** Charlotte Lauridsen, Anna Amanda Schönherz, Søren Højsgaard

**Affiliations:** 1 Department of Animal Science, Aarhus University, Blichers Alle 20, Foulum, P.O. Box 50, 8830 Tjele, Denmark; anna.schonherz@anis.au.dk; 2 Department of Clinical Medicine, Aalborg University, Mølleparkvej 4, 9000 Aalborg, Denmark; 3 Department of Mathematical Sciences, Aalborg University, Skjernvej 4A, 9210 Aalborg Øst, Denmark; sorenh@math.aau.dk

**Keywords:** vitamin E, selenium, sows, piglets, immunity, antioxidant status

## Abstract

This study investigates two levels of dietary selenium (Se) and vitamin E in combination on their status in sows and their progeny, and influence on antioxidant status and immunological responses of the piglets at weaning. Female pigs (*n* = 6) were provided LOW or HIGH antioxidant nutrition (Se and vitamin E) from mating until weaning of their off-spring. The HIGH treatment elevated the concentration of Se (*p* = 0.015) and α-tocopherol (*p* = 0.023) in plasma of piglets compared with piglets of the LOW treatment. Treatments also affected the concentrations of milk and sow plasma immunoglobulins. Piglets from sows on the HIGH treatment had increased (*p* < 0.001) activity of glutathione peroxidase, lower serum levels of C-reactive protein (*p* = 0.005), haptoglobin (*p* = 0.05) and albumin (*p* = 0.05), and the number of white blood cells (*p* = 0.023) and the ratio of NEU to LYM was lower (*p* = 0.025) than in piglets from sows on the LOW group. Furthermore, the dietary antioxidant level influenced responses of cytokines (interleukine (IL) 6 (*p* = 0.007), 12 (*p* = 0.01) and 18 (*p* = 0.01)) in piglets’ plasma. In conclusion, improved antioxidant status via dietary maternal provision improves the robustness of the offspring via immunomodulatory mechanisms.

## 1. Introduction

The role of oxidative stress and protective functions of antioxidants has gained increasing interest in relation to human and animal health and immunity. However, so far only sparse research with specific focus on swine has been conducted. Newly-born piglets are at high risk of oxidative stress and are very susceptible to free radical oxidative damage due to the increase in the O_2_ partial pressure in tissues occurring as a consequence of the foetal-neonatal transition at birth [1]. Furthermore, during frequently observed enteric disease processes of young animals, oxidative stress and inflammatory reactions in the gut can be observed [2]. Young animals such as pigs pre- and post-weaning have immature intestines making them very vulnerable towards invading microorganisms, and the typical reaction to an infection is localized inflammation of the gut, which appears during the immunological reaction to cope with the pathogens. A consequence of the respiratory burst appearing in parallel with the phagocytosis is that the development of reactive oxygen species may shift the balance between antioxidants and prooxidants towards oxidative stress.

Vitamin E and selenium (Se) are established as essential nutrients given their important functions for the immune system in both livestock [3,4] and in humans [5]. There are many examples of a synergistic action between them [6], and their deficiencies result in similar clinical disease manifestations such as porcine and humane cardiomyopathies [7]. The periods of life where vitamin E and Se deficiencies in pigs are more likely to occur are in the neonate, at weaning, and during the reproductive period [8]. Vitamin E is a fat-soluble antioxidant, which protects the polyunsaturated fatty acids in the cell membrane, regulates production of reactive oxygen species and reactive nitrogen species, and modulates signal transduction as also reviewed recently in relation to gut health of pigs [3]. The role of vitamin E in modulation of immune responses in humans and the effects of vitamin E supplementation in relation to infectious diseases show a cell-specific effect of vitamin E [9]. Through its incorporation into selenoproteins, Se is involved in the regulation of oxidative stress, redox mechanisms, and other crucial cellular processes involved in innate and adaptive immune responses [4]. In pigs, the periods immediately after birth and post weaning are critical in terms of antioxidant protection, making the young piglets especially prone to oxidative stress reactions [2]. Pigs are born vitamin E deficient and with low ascorbic acid status, and the sow colostrum and milk is the main transmission vehicle of vitamin E [10] that provides the newborn piglet with the first defenses against oxidative damage. Likewise, the piglets’ Se status is also dependent of the sows’ Se status, which is influenced by the parity of the sow. Consequently, pigs of mature sows are more likely to enter the post weaning period at a lower status than the progeny of younger sows [11]. Thus, it is important to pay attention to the sow status of vitamin E and Se to ensure good progeny status of these essential nutrients. While early nutrition in the form of micronutrient supplementation to lactating sows is well-known to influence the micronutrient status of the suckling progeny, less is known regarding the influence of piglet immune function. As recently reviewed, antioxidant nutrition can play a major role for gastro-intestinal functionality and health of young pigs [3]. The hypothesis of the present study was that providing high antioxidant diets with vitamin E and Se to sows during gestation and laction would enhance the antioxidant status of suckling piglets, and improve their antioxidant status and immunity, and their robustness towards enteric infectious diseases at weaning.

## 2. Materials and Methods

### 2.1. Dietary Treatments

Two dietary treatments for sows formed the experimental design of the present study: Diet 1 (LOW) was low in antioxidants (low Se and low vitamin E), and diet 2 (HIGH) was high in antioxidants (high Se and high vitamin E). Diet 1 was formed with addition of 20 mg vitamin E (as D,L-α-tocopheryl acetate) per kg feed and without any supplementation of Se, i.e., only endogenous Se levels from the feed ingredients. Diet 2 was formed by high supplementation of 200 mg vitamin E (as D,L-α-tocopheryl acetate) and 0.3 mg Se (as organic Se) per kg feed. After preparation, the diets were analysed for the concentration of vitamin E (α-tocopherol) by high-performance liquid chromatography technique (HPLC) after saponification and extraction into heptane as described by [12]. Concentration of Se was determined by the UT2A laboratory (Pau, France) according to the method described by [13].

### 2.2. Animals

Gilts (*n* = 6) being crossbreds of Landrace and Yorkshire were selected from the herd at Aarhus University, Foulum when they showed their first heat. Dietary treatments were initiated when the animals were showing second heat, and the gilts were randomly assigned to one of the two dietary treatment groups, with each of three gilts. The gilts were inseminated with Duroc-sperm, and during gestation, gilts were housed in loose pens in their respective dietary treatment groups. On day 108 of gestation, the six pregnant gilts were moved to the farrowing unit located in the same barn facility. After birth, cross-fostering of piglets was performed in order to obtain uniform litters of an average of 8 pigs per sow (SD = 2), and diet 1 litter contained in total 26 while diet 2 contained in total 27 piglets. Piglets were suckling the sows until weaning at day 28 of age. No creep feed was provided to the piglets during the suckling period. Animals were followed by appropriate veterinary surveillance throughout the experiment, and the experiment was performed in accordance with a permission from the Danish Animal Experiments Inspectorate, Ministry of Food, Agriculture and Fisheries, Danish Veterinary and Food Administration.

### 2.3. Sampling

Samples of the colostrum (on day 2) and of mature milk (at day 11, and 25 of lactation) was obtained from each sow by hand milking after injection of oxytocin (dose 20 international units per sow). The colostrum and milk samples were frozen at −20 °C before analysis of vitamin E, Se and determination of immunoglobulin G, A, M (IgG, IgA, IgM) concentrations. Gilts/sows were blood-sampled at the beginning of the trial, and at day 84 and 108 of gestation, at farrowing, and at day 4, and day 25 of lactation. All piglets were blood sampled at day 4, 11, and 25 of age. EDTA-containing or heparinized vacutainers (Vacuette, Greiner Bio-One GmbH, Kremsmünster, Austria) were used to collect blood samples, and plasma was separated from the blood by centrifugation at 2000× *g* and stored at −20 °C until analysis or prepared as described below.

At the end of the experiment (at weaning on day 28 of age), three piglets per sow were randomly selected and were euthanized using blunt trauma, and the intestine and the liver were removed. The intestinal tract was exposed, and after emptying the small intestine, the length of the ileum was measured in order to obtain samples of the mucus layer and epithelial layer at the mid (50%) and distal (90%) part of the small intestine for gene expression analysis. The mucosal samples were obtained by gently scraping of the mucus with the soft end of a small object glass, and the remaining tissue (without the scraped mucus) was considered the epithelial tissue. Hence, ‘mucosal’ samples were a mixture of epithelium, lamnia propria and probably submucosa, while ‘epithelial tissue’ samples also included the muscle layer. Collected epithelial tissue, mucosal samples, and liver samples were placed in RNA-later and frozen at −80 °C for later analysis.

### 2.4. Laboratory Analysis

As an indicator of vitamin E status, the plasma and milk α-tocopherol concentration was analysed by HPLC as described by [14]. The concentration of total Se was determined in sow milk and piglet plasma after acid treatment and was analysed by an ICP-MS Triple Quad system from Agilent Technologies (Santa Clara, CA, USA) located at the University of Copenhagen (Frederiksberg, Denmark). The concentrations of immunoglobulins (Ig) A, G and M were measured in plasma of piglets and sows as indicators of their humoral immune status, and in the milk of sows using commercial kits (pig ELISA quantitation kit; Bethyl Laboratories, Montgomery, TX, USA). Immediately after collection of blood samples at day 25 of age from piglets, haematological parameters including total number of leucocytes, neutrophils, lymphocytes, monocytes, eosinophils, erythrocytes, haematocrit and haemoglobin was analysed in whole blood using a haematology analyser (IDEXX ProCyte Dx^®^, Westbrook, ME, USA). Plasma samples were furthermore analysed for acute phase reactants using a porcine assay for C-reactive protein (CRP), haptoglobin and albumin according to manufactory guidelines (Tridelta Developments Ltd., Kildare, Ireland). All anayses were performed using an autoanalyzer, ADVIA 1800 Chemistry System (Siements Medical Solutions, Tarrytown, NY, USA). The antioxidative enzymes gluthathione peroxidase (GSH-Px) and superoxide dismutase (SOD) were analysed using commercial kits (Cayman Chemical, Ann Arbor, MI, USA). Concentration of malondialdehyde (MDA) was measured in plasma samples to assess the level of oxidation using a commercial kit (Cell Biolabs, San Diego, CA, USA). Plasma obtained from piglets at day 4 were analysed for lactoferrin using an enzyme-linked immunosorbent assay (Lifespan Biosciences, Seattle, WA, USA). For analysis of cell-mediated immune responses, piglet sodium-heparin stabilized whole blood (1 mL) was stimulated with 50 µL E. coli lipopolysaccharide (Escherichia coli O111:B4 LPS, Sigma-Aldrich, St. Louis, MO, USA) for 2 h at 37 °C. Stimulation was stopped by transferring samples to ice, and plasma was isolated (2000× *g*, 10 min, and 4 °C) and stored at −80 °C until analysis by the porcine MilliplexMap Kit (EMD Millipore Corporation, Billerica, MA, USA) on a Luminex 100 (Bio–Rad, Richmond, CA, USA) for the following 12 cytokines: interferron-gamma (IFN-γ), tumor necrosis factor alpha (TNF-α), and the following interleukines (IL): IL-1a, IL-1b, IL-1ra, IL-2, IL-4, IL-6, IL-8, IL-10, IL-12, and IL-18. For liver, intestinal mucosal, and epithelial samples, the inflammatory mRNA abundance was quantified by real-time reverse transcription polymerase chain reactions (qPCRs) using the ViiA7^TM^ 7 Real-time PCR system (LifeTechnologies, Singapore, Singapore) and the QuantStudio Real-Time PCR software from applied bioscience (Fisher Scientific, Slangerup, Denmark). The mRNA abundance of target genes was quantified as cycle threshold (C_q_), the number of amplification cycles required to reach the detection threshold distinguishing real signals from background noise. Target genes analysed included transcription factors PTGS2 (cyclooxygenase-2 (COX-2), TGFβ1, TNF-α, NFκBeta, and two interleukins (IL-6, IL-10). Target genes of each sample were analysed in duplicates. Two endogenous controls, glyceraldehyde-3-phosphate dehydrogenase (GAPDH) and β-actin, were confirmed to be suitable housekeeping genes, as they were not affected by the diets. For each sample, the C_q_ values for the target genes were normalized by subtracting the mean C_q_ value of the two housekeeping genes (GAPDH gene and β-actin) from the C_q_ value of the corresponding target gene (∆C_q_). The ∆C_q_ values were used in the statistical analyses to compare gene expression levels of each sample.

### 2.5. Statistical Analysis

All statistical analyses were conducted using the software R (A Language and Environment for Statistical Computing, version 3.6.2, R Development Core Team (2020), Vienna, Austria; http://www.r-project.org, accessed on the 24 June 2020). Data on vitamin E, Se and immunoglobulins in plasma and milk were analysed using a linear mixed effects model including the effect of dietary antioxidant level and sampling day as fixed- and sow as a random effect. The effect of dietary antioxidant level on expression of inflammatory genes was investigated using a linear mixed effects model including dietary antioxidant level as fixed- and sow as a random effect. Differences in gene expression were investigated for each target gene (PTGS2, TGFβ1, TNF-α, NFκBeta, IL6, IL10) and tissue (ileal mucus layer 50%, ileal mucus layer 90%, ileal epithelium 50%, ileal epithelium 90%, liver), separately. In line, gene expression in samples collected from the mid and distal part of the ileum (ileum 50% vs. ileum 90%) were compared for mucus and epithelium samples separately using a linear mixed effects model with ileal position as fixed- and sow and piglet as random effects. Linear mixed model analyses were performed using the R packages *lme4* version 1.1.23 [15], *pbkrtest* version 0.5.1 [16] and *lmerTest* version 3.1.3 [17]. Model assumptions were confirmed using the Shapiro-Wilk test of normality and the Fligner-Killeen test of homogeneity of variances, both for model residuals and the model variables. Results were visualized using the R package *ggplot2* version 3.3.1 [18].

## 3. Results

Chemical analysis showed that the LOW diets contained 0.1 mg Se and 20 mg vitamin E (α-tocopherol) per kg feed. The HIGH diets contained 0.4 mg Se and 147 mg α-tocopherol per kg feed.

### 3.1. Sows’ Concentrations of Antioxidants and Immunoglobulins

The initial vitamin E (α-tocopherol) concentration for all gilts were 1.8 mg/L plasma (SEM = 0.3) at the beginning of the experiment. The vitamin E (α-tocopherol) concentration in plasma of the animals after supplementation were 2.32 mg/L (SEM = 0.52) and 1.44 mg/L (SEM = 0.48) for the HIGH and the LOW treatment, respectively, when samples were pooled from day 84 of gestation until d 25 post farrowing. Vitamin E (α-tocopherol) and Se concentrations in milk samples of sows were significantly (*p* < 0.001) influenced by dietary treatment of sows (*p* < 0.001) and the time of sampling (*p* < 0.001). Sows on the HIGH treatment had higher colostrum and milk concentration of vitamin E than sows on the LOW treatment, irrespectively of sampling time. Moreover, colostrum contained much more vitamin E than milk samples (Table 1). Although the concentration of Se seemed to be higher in colostrum and milk of sows on the HIGH treatment, no statistically significant effects of dietary treatment or day of sampling were obtained. The concentration of all measured immunoglobulins was influenced by time of sampling (*p* < 0.001), and was in general highest in colostrum than later in the suckling period (Table 1). The concentration of IgA was higher (*p* < 0.011) in colostrum and milk of sows provided LOW, whereas the concentration of IgM was higher (*p* < 0.028) for sows on the HIGH treatment (Table 1).

### 3.2. Piglets’ Plasma Concentrations of Antioxidants and Immunity

Piglets suckling sows provided the HIGH treatment had higher vitamin E (α-tocopherol) concentrations than piglets suckling sows on the LOW treatment (*p* = 0.023), and the vitamin E concentration was higher at day 4 of age than at subsequent sampling times during the suckling period (*p* < 0.001) as shown in Table 2. Piglets suckling sows on the HIGH treatment also had higher Se concentration in plasma than piglets suckling sows on the LOW treatment (*p* = 0.015). No differences between piglets days of age were obtained with regard to the Se concentration in plasma. Correlation between plasma concentration of vitamin E and Se in piglets plasma could not be obtained.

The day of age at sampling had strong effect on the concentrations of plasma immunoglobulins in piglets, as immunoglobulin concentrations were higher at day 4 of age than later during the suckling period (Table 2). The only effect of dietary treatment on plasma immunoglobulins was obtained for the concentration of IgA (*p* = 0.045) at day 4 of age, with piglets suckling sows on the LOW treatment showing a higher IgA concentration than piglets suckling sows on the HIGH treatment. The concentration of lactoferrin measured in plasma of the piglets at day 4 were 84.9 ng/mL (SD = 30.8) and 89.6 ng/mL (SD = 9.85) for piglets suckling sows on the LOW and the HIGH treatment, respectively.

On day 25, blood was sampled for additional analysis of oxidative status and immunity. The GSH-Px activity (Table 3) was higher (*p* < 0.001) in piglets of sows provided the HIGH treatment in comparison to the piglets of sows provided the LOW treatment. The dietary treatments of sows had no influence on the activity of SOD or the MDA concentration (Table 3). The number of white blood cells (WBC) and percentage of neutrophils (NEU) was higher, whereas percentage of lymphocytes (LYM) was lower in piglets of sows provided the LOW treatment, and overall the ratio of NEU to LYM was higher in this treatment group (Table 4). Other hematological parameters were not significantly influenced by the dietary treatments of the sows. In general, an acute phase reactant response was obtained in all plasma samples obtained at day 25 of age, and provision of the LOW treatment to the sows elevated the CRP, haptoglobin and albumin concentrations in the piglets when compared to piglets of sows provided the HIGH treatment (Table 5). Furthermore, piglets suckling sows provided the HIGH treatment had higher concentrations of IL-6 (*p* = 0.009) and IL-12 (*p* = 0.016) in LPS-stimulated plasma, while the concentration of IL-18 was lower (*p* = 0.018) than in piglets suckling sows of the LOW treatment (Table 4).

### 3.3. Expression of Inflammatory Genes in Intestine and Liver

**Figure 1:**
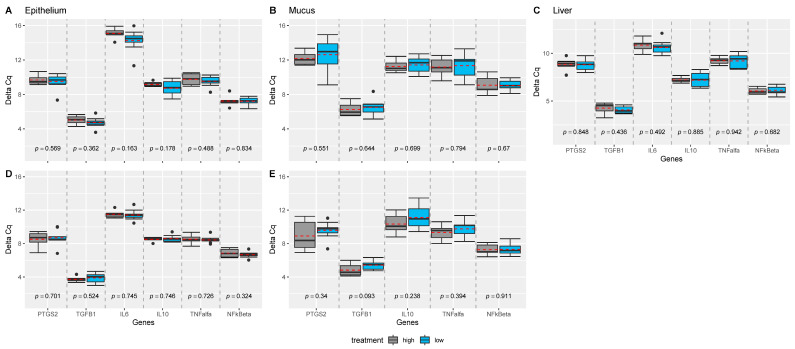
Comparison of treatment effect investigated for each gene, tissue, and ileal sample position, separately. *p*-values were estimated from mixed effect models with treatment as fixed and sow as random effect using the Satterthwaite’s method. Linear mixed effect model analyses were performed for each gene, tissue, and sample position within tissue, separately, and grouped boxplots including all genes were generated: for (**A**) epithelium samples collected from 50%; (**B**) mucosal samples collected from 50%; (**C**) liver samples, (**D**) epithelium samples collected from 90%; (**E**) mucosal samples collected from 90%. Pairwise comparisons are separated by grey dashed lines. Mean values per group are indicated by red dashed lines. *n* = 18 piglets.

**Figure 2:**
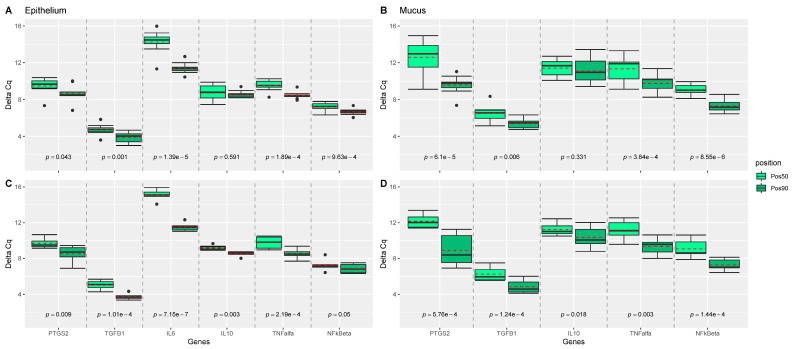
Comparison of ileal position effect (pos50 versus pos90) of epithelium and mucosal samples for each gene. *p*-values were estimated from mixed effect models with position as fixed and sow and piglet as random effect using the Satterthwaite’s method. Linear mixed effect model analyses were performed for each gene, tissue, and treatment group, separately, and grouped boxplots including all genes were generated: for (**A**) epithelium samples from the LOW treatment group; (**B**) mucosal samples from the LOW treatment group; (**C**) epithelium samples from the HIGH treatment group; (**D**) mucosal samples from the HIGH treatment group. Pairwise comparisons are separated by grey dashed lines. Mean values per group are indicated by red dashed lines. *n* = 18 piglets.

Expression of IL-1b, IL-8, and IL-17a were not measurable in the liver and intestinal samples, and expression of IL-6 was not measurable in mucus obtained at the site 50% of the length of the ileum. TNF-α was not expressed in all samples. While no effect of dietary treatments was observed on the measured genes (Figure 1), position of the sampling (e.g., 50% versus 90% of the ileal length) significantly influenced the measured expression of the genes in epithelium, and mucus of the intestine (Figure 2). For almost all genes significantly expressed (all but IL10), genes were more upregulated at the 50% site compared with the 90% site in both epithelium and mucus.

## 4. Discussion

Weaning of piglets generally increases the risk of gastrointestinal infections, and especially E. coli associated diarrhoea is frequently observed after weaning of the pigs. The post weaning diarrhoea (PWD) poses a great economic burden for pig farmers, while simultaneously accelerating the risk of antibiotic resistance development, because of the high consumption of antibiotics and medicinal zinc oxide (ZnO) for the treatment of PWD in pigs. In order to explore alternatives to antibiotics and ZnO, it is recommended to consider vitamins due to their well-described mechanisms of importance for gut development and function [3]. Se, through roles of selenoproteins, affects different types of immune responses in different ways as evidenced from studies in human and other animal species [4]. With this knowledge, the present study aimed on investigating the effect of high vitamin E and Se supplementation to sows on piglets’ antioxidant status and immunity.

The dietary level of vitamin E and Se in the HIGH antioxidant treatment was considerably higher than the official nutritional recommendation for pigs [19], in which reproducing sows should be provided 40 mg of vitamin E and 0.2 mg/kg of Se per kg diet. Overall, the present experiment showed a clear influence of the dietary vitamin E and Se provided to the sows on subsequent plasma concentrations of these antioxidants in the piglets. These results confirm previous studies in pigs on Se [20,21] and vitamin E [22], showing that dietary supplementation of vitamin E and Se elevates the plasma status of these micronutrients in sows and their offspring.

Colostrum (obtained at day 2 after farrowing) had a higher α-tocopherol content than milk sampled later during the lactation period, and the content was elevated due to the supplemented dietary vitamin E as also observed by [22]. The obtained Se levels in colostrum and milk were in accordance with contents measured in colostrum and mature milk of sows by [23]. Previous studies in dairy cattle [24], and in sows [23] have shown that colostrum contains considerably more Se than milk. The limited number of sows in the present experiment and the large variation in Se-content was probably the reason why no statistical effect of treatments or time could be obtained with regard to the Se concentration in colostrum and milk. However, when pigs were suckling sows provided the high dietary level of vitamin E and Se, a clear effect on plasma concentration in piglets were obtained hence indicating the high bioavailability of the vitamin E and Se in colostrum and sow milk to the nursing pig.

This was confirmed by measuring the activity of GSH-Px in piglets’ plasma at day 25 of age, which was increased when sows were supplemented with the HIGH treatment. It is well-known that dietary Se provision influences the activity of Se-dependent GSH-Px in pigs [25]. Thus, dietary vitamin E and Se supplementation to sows influenced antioxidant status of pigs assessed by the concentration of α-tocopherol, Se and activity of GSH-Px in plasma, meaning that the pigs entered the post weaning period with clear differences in the status of these antioxidants. However, we observed no effect on the antioxidant status measured in terms of MDA concentration and SOD activity in the suckling pigs of sows provided the different dietary antioxidant levels. In previous studies by [26,27], effects of two Se sources provided at the level of 0.30 mg Se/kg diet, and two vitamin E levels (at 30 and 90 IU/kg diet) for gestating and lactating diets of sows were investigated with regard to antioxidant status of sows and their progeny. While no effects of these dietary treatments were obtained on SOD in liver and serum, the MDA serum concentration was lower in the pigs provided the organic rather than inorganic Se-source, and no effect was obtained on the MDA concentration in the liver [27]. However, when measured in sows serum and milk, SOD was affected by the Se form [26], whereas the vitamin E level in general had less impact on the measured antioxidant status in sows and their offspring [26,27]. According to these studies, the lack of influence on SOD and MDA concentration in piglets of our study was not surprising.

The influence of the combination of vitamin E and Se on immunological responses of pigs has been investigated before, however, only little information is available. Peplowski et al. [28] showed that weanling-grower pigs either injected or fed both nutrients (vitamin E and Se) had a higher humoral antibody response than when either nutrient was provided independently. A more recently published study on growing pigs revealed that an increase in dietary Se and vitamin E mitigated the impacts of heat stress on intestinal barrier integrity, associated with a reduction in oxidative stress [29]. In the present study, we investigated both the passive immunity transfer, and the humoral and intestinal immune responses of the pigs. An adequate passive transfer of maternal immunoglobulins to the newborn piglet is paramount as the porcine placenta act as a barrier for the transport of maternal immunoglobulins to the fetus [30]. As expected, the colostrum had a much higher content of IgG and IgM than milk as also shown in a recent study on sows from the same herd [31]. Interestingly, the sows provided the high antioxidant supplementation in the present study had an increased concentration of IgM in colostrum and milk. Furthermore, an additive relationship between the content of α-tocopherol or IgG in milk and the effect of treatment was obtained. Overall, these results could be interpreted as enhanced passive immune provision when sows are provided high antioxidant level. However, the concentration of IgA in notably sows’ colostrum and piglets’ plasma at day 4 of age was higher at the low antioxidant treatment. The maternal IgA supports the development of the gut function in pigs, and IgA contributes with a higher proportion of the total immunoglobulins in milk than in colostrum. During the first few hours after birth, the piglet’s intestine is able to absorb macromolecules in a non-selective way. However, a rapid ‘closure’ of the gut for macromolecule uptake happens 24 to 48 h after birth [32]. This may explain the increased concentrations of IgA in plasma of piglets at day 4 from sows provided low antioxidant supplementation. Analysis of plasma lactoferrin, which is an important modulator of immune function and is involved in the host defence response against bacteria, was not influenced by the dietary treatments. However, in piglets of two sows on the LOW antioxidant treatment, the lactoferrin concentration in the piglet plasma on day 4 was higher than in piglets from sows on the HIGH antioxidant treatment. It cannot be excluded, that the sows, which were genetically susceptible to *E. coli,* were exposed to *E. coli* during the experimental period, and that their immune responses, which were influenced by their dietary treatments, were reflected in the colostrum and milk.

In line, the measured acute phase response in all piglets at day 25 of age may indicate that an inflammatory response was elicited. Values for CRP and haptoglobin were in the same range as observed for pigs studied in an infectious study carried out in the same experimental facility [33]. The concentration of haptoglobin was in general considerably higher when compared to the values obtained in the study by [34] investigating the effect of different levels and forms of dietary Se in heat stressed growers. Although the measured acute phase responses in general may reflect a non-specific immunologic response (demonstrating changes similar to infection in other inflammatory condition) [35], it supports the explanation above that animals were exposed to a pathogenic challenge in the suckling environment. Interestingly, piglets from sows provided the LOW antioxidant treatment were affected the most, as these piglets had higher concentrations of CRP, haptoglobin, and albumin, and higher number of white blood cells, and a higher ratio of neutrophils to lymphocytes. In other words, our study indicated that high antioxidant provision to the piglets via the sows’ milk benefitted the piglets as the measured acute inflammatory responses were reduced meaning that the piglets with the high antioxidant status were more capable to resist the challenge.

Furthermore, the influence of dietary treatments on piglets’ plasma cytokine responses measured just prior to weaning indicated a role of the dietary antioxidant treatments for the immunological reactions. Vitamin E and Se can modulate lymphocyte differentiation and proliferation [5]. Vitamin E protects cell membranes from damage caused by free radicals and supports integrity of epithelial membranes; maintains or enhances NK cell cytotoxic activity, inhibits PGE_2_ production by macrophages and thus indirectly protects T-cell function [5]. Se is essential for the function of selenoproteins, which act as redox regulators and cellular antioxidants and are thus important for the function of leukocytes and NK cells [36]. LPS-induced inflammation response in pigs impaired the metabolism of Se and was associated with dysregulation of the selenogenome expression in immune tissues [37]. While no difference between dietary treatments was obtained with regard to frequently reacting cytokines induced during an inflammatory response, e.g., INF-γ and TNF-α, concentrations of the cytokines IL-6, IL-12 and IL-18, which have a relationship as the pro-atherogenic cytokines in human [38], were affected by dietary treatments. IL-6 can act as a pro-inflammatory cytokine and an anti-inflammatory myokine, and may indicate a systemic inflammation. IL-12 is a cytokine, which is produced by various immune cells upon antigenic stimulation. Thus, the higher concentrations of IL-6 and IL-12 in piglets suckling sows provided the HIGH antioxidant treatment might indicate better immunological responsiveness hence reducing the level of inflammation indicated by the lower CRP and haptoglobin. IL-18 can induce cell-mediated immunity following infection with microbial product such as LPS. IL-18 in combination with IL-12 acts on CD4, CD8 T cells and NK cells to induce INF-γ production type II interferon that plays an important role in activating the macrophages or other cells. Thus, the higher concentration of IL-18 in plasma of piglets of sows provided the low antioxidant supplementation may indicate immunological activity provoked by the E. coli LPS stimulation of the blood.

We furthermore studied the impact of the dietary antioxidants on tissue inflammatory genes. Oxidative stress may compromise intestinal epithelial barrier integrity in pigs upon challenges such as heat stress [29]. High Se (1.0 mg/kg) and vitamin E (200 mg/kg) reduced intestinal leakiness caused by heat stress in growers when compared with low dietary antioxidant levels (0.2 mg/kg Se and 17 mg vitamin E/kg), but did not alter intestinal inflammatory (IL-8 and TNF-α) mRNA expression [29]. In accordance, no effects of dietary treatments in our study were obtained with regard to the expression of genes in the intestinal mucus and epithelium and in the liver of piglets. Although we did not measure the concentrations of Se and vitamin E in these tissues of the pigs, it would be expected according to previous studies [21,22,23,34] that the progeny tissue concentrations of these micronutrients would be enhanced by dietary supplementation levels of the sows. However, the difference between sampling sites in expression of the measured genes in mucus and epithelium indicated a variation due to physiological factors, which overwhelmed the potential influence of the dietary treatments. The fact that no expression differences between the dietary antioxidant level were obtained for any of the inflammatory genes, and that the expression of TNF-α was not measurable in all intestinal samples, generally support why no difference could be obtained with regard to the concentration of the pro-inflammatory cytokines, such as INF-γ and TNF-α, measured in the plasma.

## 5. Conclusions

To summarize, our results indicate that the elevated antioxidant status of piglets during the suckling period obtained by high dietary vitamin E and Se supplementation of sows enhanced piglets’ cell mediated immune responses and reduced inflammatory responses at weaning. Overall, the improved antioxidant status prior to weaning may improve the robustness of the pigs via immunomodulatory mechanisms, which may benefit the piglet upon challenges appearing during the transition period of weaning.

## Figures and Tables

**Table 1:** Effect of dietary antioxidant level ^1^ on concentration of vitamin E (α-Toc.), selenium (Se) and immunoglobulins (Ig) A, G, and M in colostrum (day 2) and milk of sows (*n* = 3 sows per treatment. Means and standard error of mean (SEM)).

Time after Birth	Day 2		Day 11		Day 25		*p*-Value	
	LOW	HIGH	LOW	HIGH	LOW	HIGH	Treatment	Time
α-Toc, mg/L ^1^	6.64 (3.00)	24.2 (3.72)	2.07 (0.23)	6.53 (0.26)	1.37 (0.21)	4.14 (0.48)	<0.001	<0.001
Se, μg/L	74.5 (38.8)	112 (4.3)	66.5 (18.2)	77.9 (21.6)	53.7 (22.8)	89.9 (32.3)	0.15	0.54
IgA, mg/L	3829 (1688)	2921 (812)	3157 (523)	2775 (276)	2604 (360)	2892 (324)	0.011	0.20
IgG mg/L	12,615 (10,500)	7498 (4867)	474 (96.2)	662 (72.3)	209 (54.6)	388 (22.9)	0.47	<0.001
IgM, mg/L	2650 (53.7)	4066 (1259)	2266 (111)	4434 (1128)	1188 (107)	2996 (631)	0.028	0.158

^1^ When data were analysed on a logarithmic scale, the visual analysis revealed an additive relationship between the content of α-tocopherol or IgG and the effect of treatment.

**Table 2:** Effect of maternal dietary antioxidant level on concentration of vitamin E (α-Toc.), selenium (Se) and immunoglobulins (Ig) A, G, and M in plasma of off-springs (LOW: *n* = 26 piglets; HIGH: *n* = 27 piglets; means and standard error of mean (SEM)).

Time after Birth	Day 4		Day 11		Day 25		*p*-Value	
	LOW	HIGH	LOW	HIGH	LOW	HIGH	Treatment	Time
α-Toc, mg/L	3.56 (0.41)	6.39 (0.62)	1.61 (0.12)	4.65 (0.28)	2.26 (0.14)	5.41 (0.31)	0.023	<0.001
Se, μg/L	11.6 (1.10)	19.9 (2.73)	7.38 (0.98)	21.1 (1.17)	9.41 (0.75)	20.5 (1.13)	0.015	0.67
IgA, mg/L	2477 (226)	1256 (133)	202 (29.1)	146 (15.1)	113 (14.1)	127 (9.68)	0.045	<0.001
IgG, mg/L	18483 (1672)	15497 (1283)	12275 (1262)	9455 (756)	4981 (346)	4272 (380)	0.26	<0.001
IgM, mg/L	1332 (100)	1816 (138)	353 (33.0)	515 (32.3)	806 (59.2)	898 (78.9)	0.16	<0.001

**Table 3:** Effect of maternal dietary antioxidant level on activity of antioxidative enzymes in plasma of off-springs at day 25 of age (LOW: *n* = 26 piglets; HIGH: *n* = 27 piglets; Mean and standard error of mean (SEM)).

	LOW	HIGH	SEM	*p*-Value
GSH-Px, nnmol/min/mL	256	338	60.4	<0.001
SOD, U/mL	0.63	0.78	0.24	0.174
MDA, μM	18.0	18.3	4.11	0.794

**Table 4:** Effect of maternal dietary antioxidant level on haematological parameters of the off-springs at day 25 of age (LOW: *n* = 26 piglets; HIGH: *n* = 27 piglets; Mean and standard error of mean (SEM)).

	LOW	HIGH	SEM	*p*-Value
WBC, ×10^9^/L	19.3	14.3	7.28	0.027
Neutrophils, %	11.7	7.4	6.57	0.031
Lymphocytes, %	37.2	53.3	16.4	0.002
Monocytes, %	3.36	3.77	1.50	0.361
Eosinophils, %	1.08	0.95	0.65	0.482
Erythrocytes, ×10^12^/L	6.25	6.42	0.47	0.220
Haematocrit, %	37.5	39.4	3.38	0.073
Haemoglobin, mmol/L	113	117	10.2	0.147
Neutrophils:Lymphocytes	1.81	1.16	0.94	0.025
Reticulocytes, %	4.24	5.28	2.34	0.145

**Table 5:** Effect of maternal dietary antioxidant level on C-reactive protein (CRP), haptoglobin, albumin and cytokines (after LPS-stimulation) in plasma of off-springs at day 25 of age (LOW: *n* = 26 piglets; HIGH: *n* = 27 piglets; Mean and standard error of mean (SEM)).

	LOW	HIGH	SEM	*p*-Value
CRP, µg/mL	767	300	489	0.005
Haptoglobin, mg/mL	1.34	0.84	0.79	0.05
Albumin, g/L	34.2	32.0	3.38	0.05
INF, ng/mL	3.37	2.97	1.73	0.49
IL-1a, ng/mL	0.21	0.21	0.13	0.96
IL-1b, ng/mL	5.04	3.97	3.33	0.33
IL-1ra, ng/mL	4.30	4.76	3.70	0.71
IL-2, ng/mL	0.04	0.05	0.022	0.63
IL-4, ng/mL	0.06	0.13	0.23	0.37
IL-6, ng/mL	0.59	1.01	0.44	0.007
IL-8, ng/mL	0.84	0.85	0.79	0.99
IL-10, ng/mL	0.16	0.15	0.07	0.64
IL-12, ng/mL	0.77	1.24	0.52	0.01
IL-18, ng/mL	0.69	0.48	0.25	0.02
TNF-α, ng/mL	0.26	0.31	0.30	0.60
INF:IL-4	67.2	56.6	39.9	0.42

## Data Availability

The data presented in this study are available on request from the corresponding author.
